# Effectiveness of Sensory Integration Therapy on Functional Mobility in Children With Spastic Diplegic Cerebral Palsy

**DOI:** 10.7759/cureus.45683

**Published:** 2023-09-21

**Authors:** Vaishnavi B Warutkar, Rakesh K Kovela, Snehal Samal

**Affiliations:** 1 Physiotherapy, Ravi Nair Physiotherapy College, Datta Meghe Institute of Higher Education and Research (Deemed to be University), Wardha, IND; 2 Physiotherapy, Nitte Institute of Physiotherapy, Nitte (Deemed to be University), Mangalore, IND

**Keywords:** short sensory profile, sensory integration therapy, pediatric-mmse, gmfcs, cerebral palsy

## Abstract

Background

A set of non-progressive brain abnormalities and nervous system dysfunctions are referred to as cerebral palsy (CP). Due to this, the child's mobility, eyesight, learning, and thought processes are affected. It can evolve before, through birth, or the first year of a child's life. The activity through which the brain organizes and analyses external sensations like touch, motion, body awareness, vision, hearing, and gravity is indicated as sensory integration. The use of sensory integration therapy (SIT) necessitates that the sensorimotor exercises target the specific parts of difficulties that the child experiences daily. This study aims to study the effectiveness of SIT on functional mobility in children with spastic diplegic CP.

Methods

In this study, 40 children of CP with spastic diplegic who met the inclusion and exclusion criterion were enlisted and were separated into two groups, with Group A (n=20) receiving SIT for 25 minutes along with conventional physiotherapy for 20 minutes, and Group B (n=20) were given conventional physiotherapy for 45 minutes. A four-week therapy plan was followed. Short sensory profile (SSP) and Gross Motor Function Classification System (GMFCS), Pediatric mini-mental state examination (MMSE), and Modified Ashworth Scale were taken as outcome measures.

Results

SIT along with traditional treatment is described in the study protocol which aids CP children to improve themselves. Following a four-week protocol, combined therapy of SIT and conventional physiotherapy show an effect on the motor function of the children. After therapy, scores in GMFCS and SSP improved. By using Student’s paired t-test, a statistically significant difference was found in GMFCS score at pre and post-test treatment in group A (7.28, p=0.0001) and group B (4.48, p=0.0001), in SSP score at pre and post-test treatment in group A (27.91, p=0.0001) and group B (11.31, p=0.0001), in MMSE score at pre- and post-test treatment in group A (6.89, p=0.0001) and group B (6.32, p=0.0001). The significance threshold was p<0.0001.

Conclusion

Under the study's experimental conditions, both groups showed substantial improvements in the functional mobility of children. When the efficacy of SIT along with conventional physiotherapy was examined, the impact resulted in a significantly greater improvement in the functional mobility of spastic diplegic CP children.

## Introduction

Cerebral palsy (CP) is a group of non-progressive disorders of the brain and nervous system dysfunction. It influences the child's mobility, eyesight, learning, and thought process. It can develop before, during birth, or the first year of a child's life [1]. As a result of brain damage and defects, it can occur two years after birth. It appears in certain children as a result of cerebral hypoxia, and preterm infants are more prone to get CP [2]. The development of mobility and posture is impacted by CP, which also limits activities [3]. Children with CP are limited in their everyday regular physical activity due to motor dysfunction [4]. Among the major objectives of therapy for individuals with CP is just to develop movement function. The application of various neurorehabilitation approaches is necessary for modification to change the functional status [5]. There is research indicating that functional treatments based on activity augmentation are beneficial in raising the performance level of pediatric CP patients [6,7].

The incidence of CP has been estimated to be 1.5 to 2.5 per 1,000 childbirths. Between 1960 and 1980, these rates were greater. Data now shows a rate of 0.5 per 1,000 births [1]. The survival rate with diplegic CP is 95% compared to quadriplegic children which is 75% up until 30 years of age. Mild to severe mental retardation has a survival rate of 65% and 90% until 38 years of age, respectively, with an overall survival rate of 90% until 20 years of age [8]. CP is classified depending on the affected limbs (hemiplegia, diplegia, or tetraplegia) and also the signs of neurologic disorders (spasticity, hypotonicity, dystonic, athetonic, or a combined impairment). Some signs differed based on gestational age at birthing, chronologic age, division of lesions, and other pathology [9]. Spastic diplegia is a kind of CP that is frequent in preterm children, particularly the most immature preterm children. During the first four months, most newborns with spastic diplegic CP have a usual tone or even hypotonia. The emergence of spasticity in the legs is gradual and progressive throughout the first year [10].

Ayres was indeed an OT (occupational therapist) and neuropsychologist who dedicated her professional life to doing research, creating remedial theories, and treating people with developmental and social difficulties. To inform her knowledge of formerly unresearched sensory (and motor) deficiencies impacting learning and performance, Ayres drew significantly on the neuroscience study. The early study by Ayres, who studied under Margaret Rood, focused on how proprioception might help with the bodily activity required for daily tasks [11]. Jane Ayres, in 1972, defines Sensory Integration as “the Neurological process that organizes sensation from one's own body and the environment, allowing the body to function effectively within the environment.” The proprioceptive, vestibular, and tactile systems were central to Ayers' idea. The activity through which the brain organizes and analyses external sensations such as touch, motion, body awareness, vision, hearing, and gravity is referred to as sensory integration. It happens in the brain and gives fine stability among the central as well as peripheral nervous structures, and also the excitatory and inhibitory neural structures [12]. Brushes, swings, trampolines, balls, and other toys that cause proprioceptive, tactile, and vestibular difficulties serve as stimuli during activity [13,14]. To increase arousal states, exercises might also include deep pressure, joint compression, oral moral exercise, and body massage [15]. As a result, sensory integration is introduced into play. The use of SIT necessitates that the sensorimotor exercises target the specific areas of difficulties that the child experiences daily. Activities often target many sensory systems simultaneously and activate skin-based proprioceptors, inner ear receptors, hearing, vision, and touch receptors as well as proprioceptors in joints and muscle groups. Results of the treatments are frequently gathered, and the intervention plan is modified as necessary [14]. Sensory integration explains the process by which the nervous system turns sensory data into action. The limbic system, as well as the vestibular and proprioceptive mechanisms, may be impacted by registration and modulation issues [15,16]. The sensory data from bodily movement across the area is handled by the vestibular system. According to Ayres' theory, the vestibular system controls the way one may respond to a stimulus, and the vestibular nuclei register and interpret visual information [16,17]. SIT is a rehab-based method that emphasizes the therapist-child bond and makes use of game-based sensory and motor activities intended to enhance the interpretation and incorporation of sensations [18,19].

The neuroplasticity at the core of SIT treatments aims to mold the neural system via experience. Because of the experiences given during the therapy, guided participation in sensory-motor tasks included in play can produce neuroplastic reactions that lead to adaptive behaviors [11]. When comparing uni- and bilateral neuroimaging patterns, using the impairment index (18), Being able to walk (GMFCS I-II), having an Intelligence Quotient (IQ) of 70 or higher, having no vision and hearing impairment, or seizure are all considered with low impairment. Unable to walk (GMFCS IV-V) and/or serious intellectual disability (IQ < 50), with or without one or more of the given impairments: severe vision issues, severe hearing issues, and active seizure, are considered under high impairment. All other levels of impairment that are not classified as low or high are classified as moderate impairment [20]. Children with spastic diplegia have noteworthy limitations in their everyday activities and participation. Their disease restricts their social participation, and it is also a major factor in determining their standard of living. The activity and involvement of affected children vary as per the seriousness of their disease, their age, their ability to self-manage, and their ability to move and function in society. It was found that there is a lack of literature indicating SIT can improve functional mobility in CP children. We hypothesized that SIT would aid in the refining of functional mobility in CP children.

## Materials and methods

Ethical clearance was issued from the Institutional Ethical Committee (IEC). The IEC approval number was DMIMS(DU)/IEC/2022/1039. The study design was a Randomized control trial. Once ethical approval was obtained children with CP were recruited from Physiotherapy OPD. The study period was six months, from September 2022 to February 2023. Inclusion criteria were the age group of six to 10, spastic diplegic CP children with GMFCS level I to III. Also, the children who can follow commands (pediatric mini-mental status examination (MMSE) score >23). Exclusion criteria were children with mental retardation and learning difficulty and children having uncontrolled seizures for the past six months or having undergone any orthopedic surgery.

A total number of 40 children were involved based on the inclusion and exclusion criterion without specific gender distribution. Proper approval was taken from the parents and was briefed about the study. Pre-treatment and post-treatment assessments were done which included short sensory profile (SSP), GMFCS, pediatric MMSE, and modified Ashworth Scale (MAS). Data were recorded and they were given the treatment for four weeks. Statistical analysis was done by using descriptive and inferential statistics using the Chi-square test, and the student’s paired and unpaired t-test and p<0.05 is observed as the level of significance.

Intervention

In every session, activities and the following exercises were done by the groups.

Group A

The experimental group was given SIT for 25 minutes and conventional physiotherapy for 20 minutes, which included the following.

Vision perception activity: Block design, detecting different forms in photos/pictures, puzzles, matching geometric forms and alphabets, numeral.

Body awareness: Directing to the body part, life-size drawings, turning from one side to another side, and awareness about body parts by touching.

**Figure 1:**
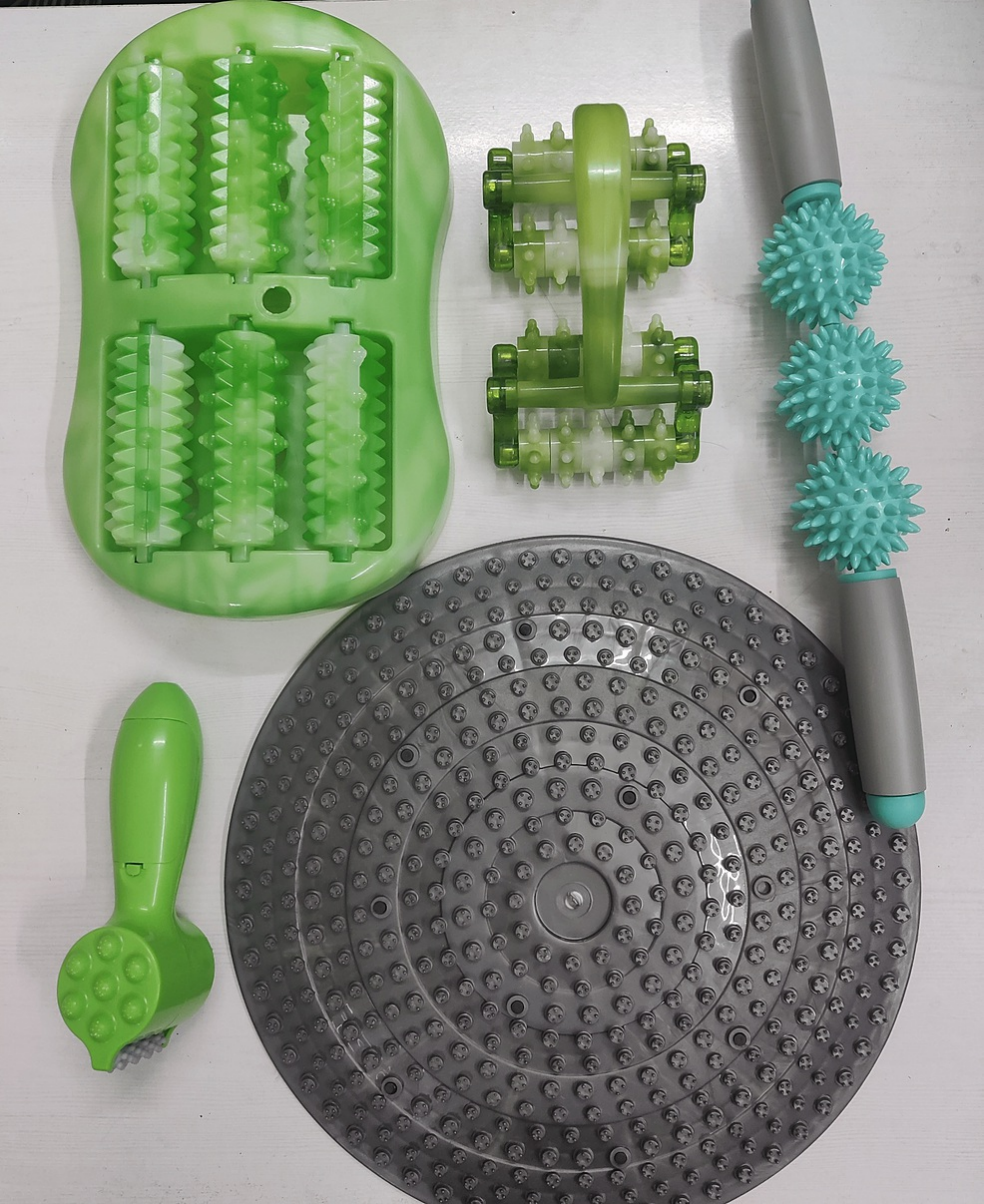
Few equipment from the sensory integration therapy kit.

Tactile perception: Feeling a variety of shapes, touching boards, and feeling textures. Figure 1 shows a few equipment from the SIT kit.

**Figure 2:**
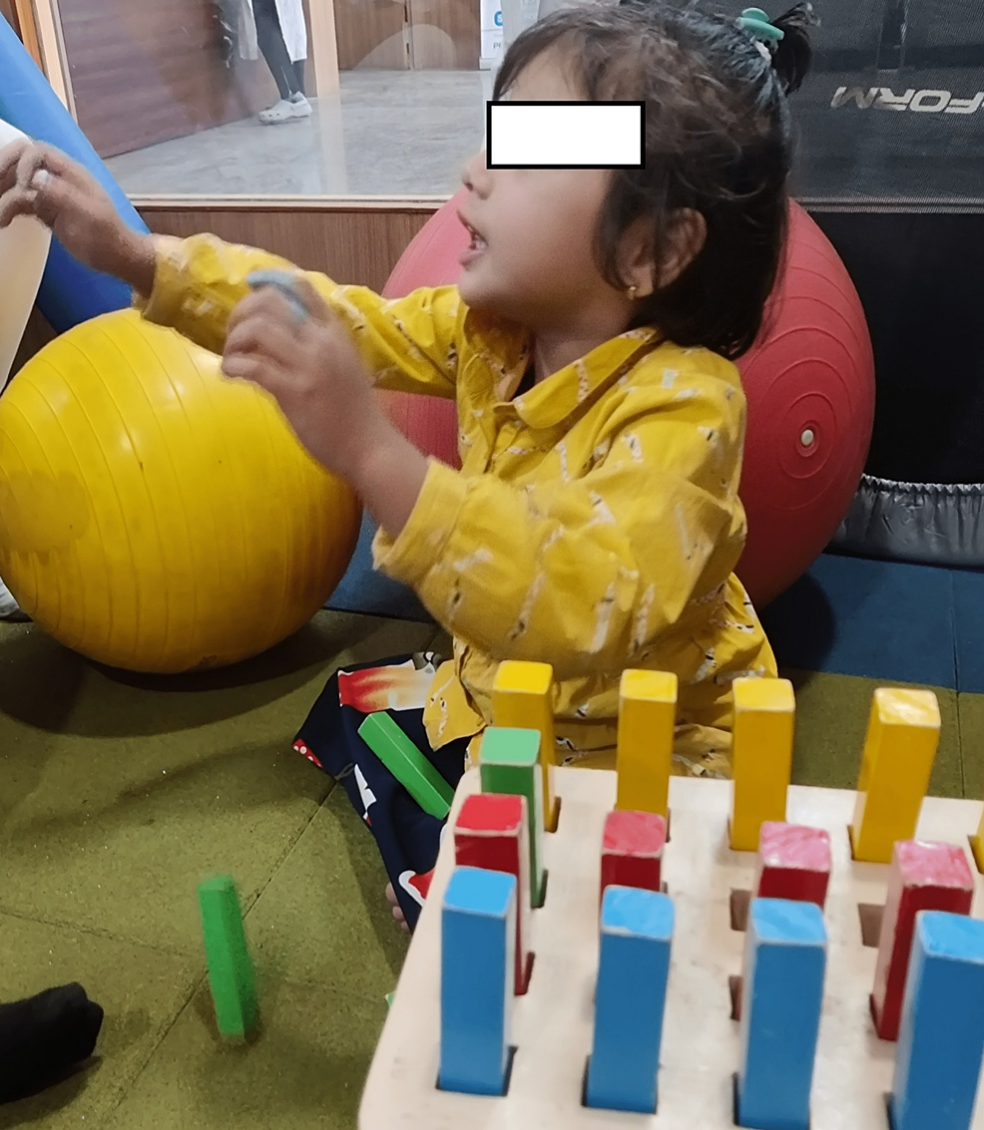
Female child doing peg board activity.

**Figure 3:**
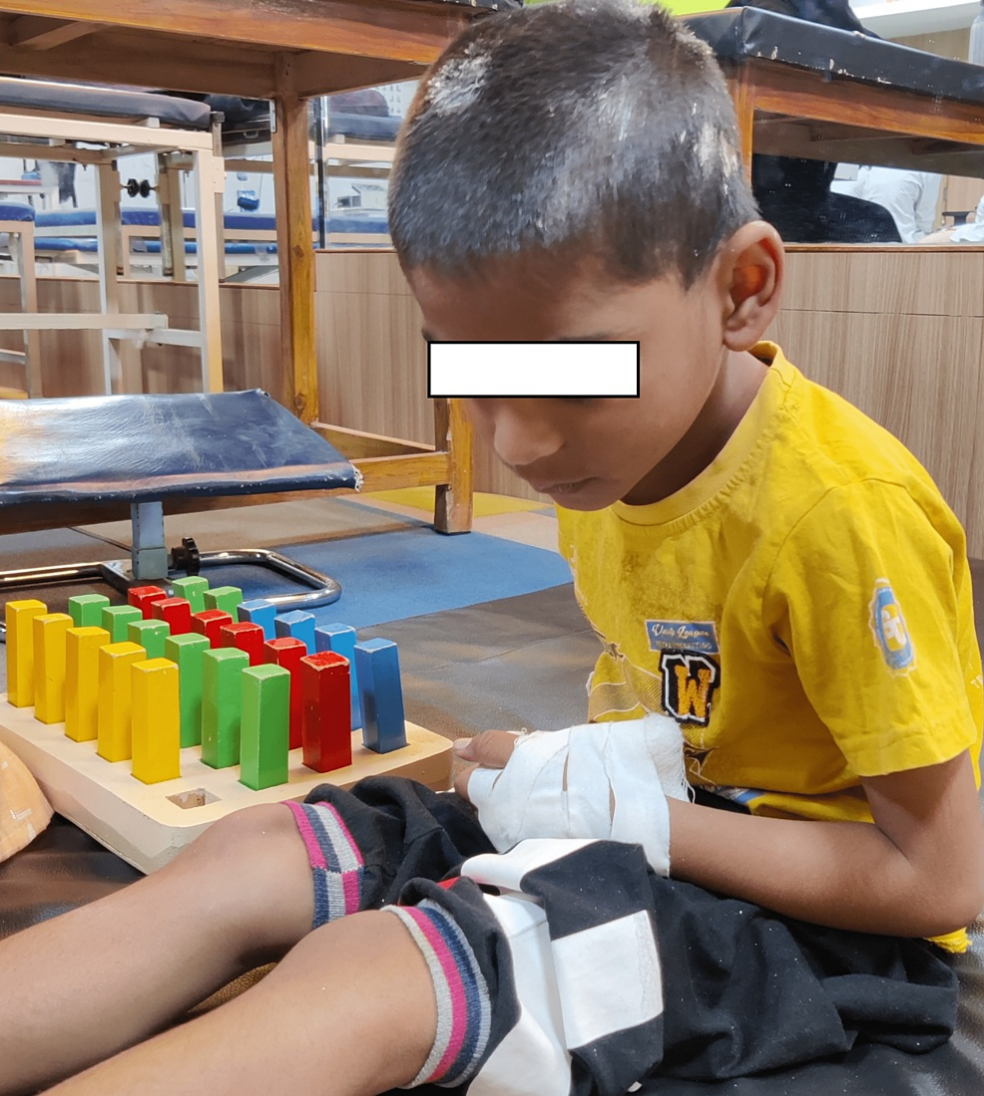
Male child doing peg board activity.

Visual-motor coordination training: Ocular-pursuit teaching, ball and pegboard playing pursuits (Figures 2, 3).

**Figure 4:**
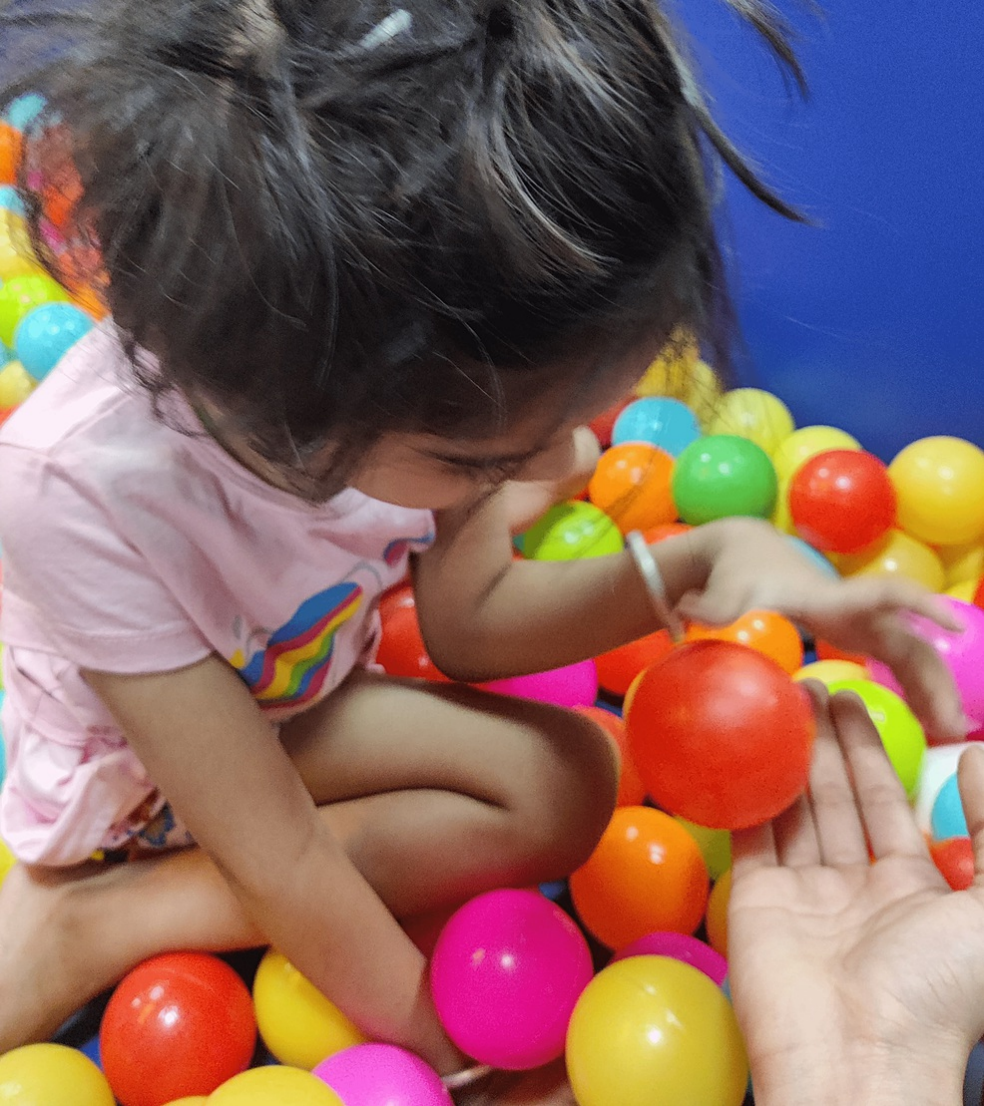
Ball catch and throw.

Proprioception: Ball squeeze, joint compression, ball catching, and throwing (Figure 4).

In every session, activities involved children sustained in sitting, on forearms and hands, crawling, semi-kneeling, and standing postures. Equilibrium and corrective responses were grown by using a Swiss ball and Wobble board after the child had obtained the skill of keeping the exercise position. Gait training, suitable for the motor development of the child (crawling, creeping, and walking in parallel bars) was given.

Group B

The control group was given Conventional Physiotherapy only for 45 minutes, which included the following. For the upper limb, lower limb, and trunk, stretching exercises, strengthening exercises, and range of motion exercises were given. Transition activities in supine lying, crawling, rolling, sitting, kneeling, standing, and walking.

## Results

Chi-square test (X2), student's paired and unpaired t-test, and software versions SPSS 27.0 (IBM Corp., Armonk, NY) and GraphPad Prism 7.0 were used for the statistical analysis, with a threshold of significance of p<0.05 being utilized for both descriptive and inferential statistics.

In this research, as per the inclusion criteria, the age group included was six to 10 years. In Table 1, it is shown that the mean age in Group A (Experimental group) was 8.20±1.36 in which, six- and seven-year-old participants were six (30%), eight- and nine-year-old participants were 10 (50%) and four (20%) were 10 years old. The mean age in Group B (Control group) is 7.40±1.39 in which the six-year-old participant was six (30%), the nine-year-old participant was one (5%), and in age groups eight and 10, a total of six (30%) were taken. In the experimental and control group X2 value was 5.91 and p=0.20.

**Table 1:** Division of children as per the age

Age (yrs.)	Group A	Group B	X2-value
6 yrs.	3(15%)	6(30%)	5.91 p=0.20, NS
7 yrs.	3(15%)	7(35%)	
8 yrs.	5(25%)	3(15%)	
9 yrs.	5(25%)	1(5%)	
10 yrs.	4(20%)	3(15%)	
Total	20(100%)	20(100%)	
Mean±SD	8.20±1.36	7.40±1.39	
Range	6-10 yrs.	6-10 yrs.	

**Figure 5:**
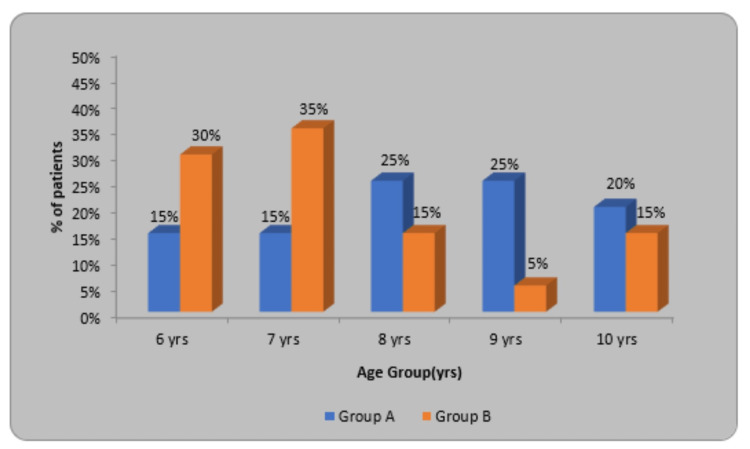
Distribution of children according to age.

Figure 5 shows the percentage of patients in a particular age group from six to 10 years. The experimental group (Group A) is denoted by blue color and the Control group (Group B) by orange color.

In Group A 50% were male and 50% were females but in Group B, 70% were male while 30% were females. In this, the X2 value was 1.66, and p=0.19 (Table 2).

**Table 2:** Division of children as per gender.

Gender	Group A	Group B	X2-value
Male	10(50%)	14(70%)	1.66 p=0.19, NS
Female	10(50%)	6(30%)	
Total	20(100%)	20(100%)	

**Figure 6:**
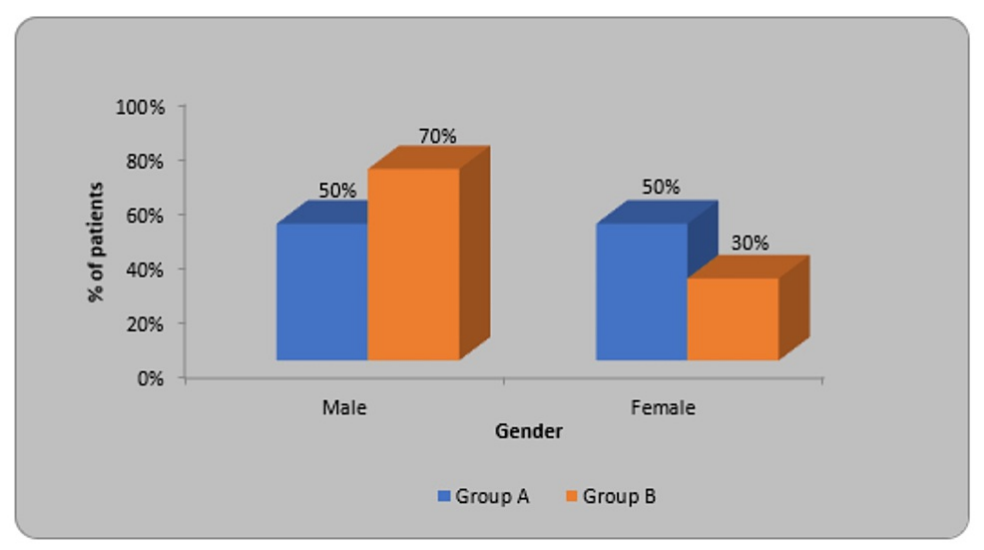
Distribution of children according to gender.

The experimental group (Group A) is denoted by blue color, and the Control group (Group B) by orange color. In Group A, males and females were equal while in Group B, males were more as compared to females (Figure 6).

The mean Gross Motor Function Classification System (GMFCS) scoring in participants of group A before treatment was 2.10±0.71 and after treatment was 1.20±0.41, in group B before treatment was 2.35±0.74 and after therapy was 1.75±0.55. By using the Student’s paired t-test, a statistically significant difference was seen in GMFCS score at pre and post-test in group A (7.28, p=0.0001) and group B (4.48, p=0.0001). On comparing the mean difference in GMFCS score in the children of both groups, statistically significant variation was seen in both groups (Table 3).

**Table 3:** Comparison of GMFCS score in two groups. GMFCS - Gross Motor Function Classification System

Groups	Pre-Test	Post Test	Student’s paired t-test
Group A	2.10±0.71	1.20±0.41	7.28, p=0.0001, S
Group B	2.35±0.74	1.75±0.55	4.48, p=0.0001, S
Student’s unpaired t-test	1.08, p=0.28, NS	3.58, p=0.001, S	

**Figure 7:**
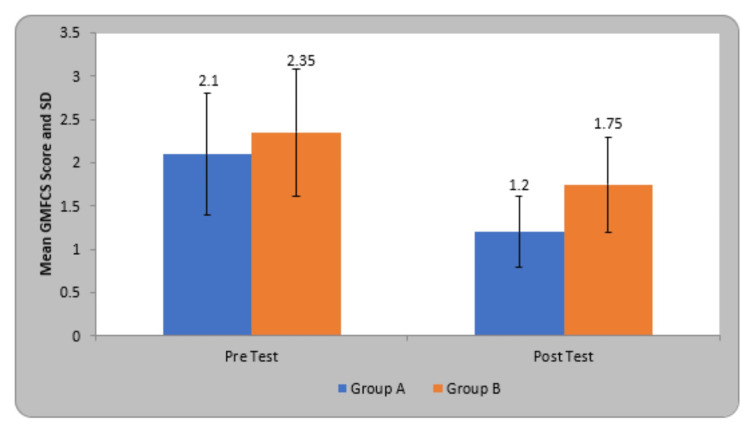
Comparison of GMFCS score in two groups. GMFCS - Gross Motor Function Classification System

The experimental group (Group A) is denoted by blue color, and the Control group (Group B) by orange color (Figure 7).

The mean SSP score in participants of group A before treatment was 127.05±3.44 and after treatment was 145.15±3.23, in group B before treatment was 117.20±8.50 and after treatment was 125.20±7.95. By using Student’s paired t-test, a statistically significant difference was seen in SSP score at pre and post-test therapy in group A (27.91, p=0.0001) and group B (11.31, p=0.0001). On comparing the mean difference in SSP scores in children of both groups, statistically significant variation was found in both groups (Table 4).

**Table 4:** Comparison of SSP score in two groups. SSP - Short sensory profile

Group	Pre-Test	Post Test	Student’s paired t-test
Group A	127.05±3.44	145.15±3.23	27.91, p=0.0001, S
Group B	117.20±8.50	125.20±7.95	11.31, p=0.0001, S
Student’s unpaired t-test	4.80, p=0.0001, S	10.38, p=0.0001, S	

**Figure 8:**
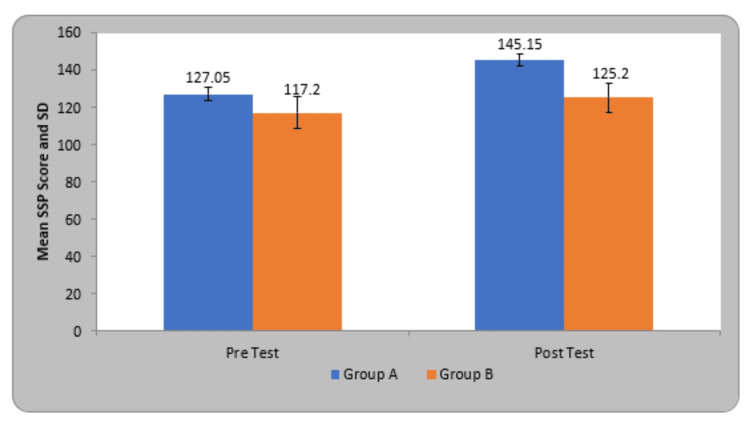
Comparison of SSP score in two groups. SSP - Short sensory profile

The experimental group (Group A) is denoted by blue color and the Control group (Group B) by orange color (Figure 8).

The mean mini-mental state examination (MMSE) score in participants of group A before treatment was 29.70±2.53 and after treatment was 32.40±2.30, in group B before treatment was 26.90±2.77 and after treatment was 28.75±2.09. By using Student’s paired t-test, a statistically significant difference was seen in MMSE score at pre and post-test treatment in group A (6.89, p=0.0001) and group B (6.32, p=0.0001) (Table 5).

**Table 5:** Comparison of MMSE score in two groups. MMSE - mini-mental state examination

Group	Pre-Test	Post Test	Student’s paired t-test
Group A	29.70±2.53	32.40±2.30	6.89, p=0.0001, S
Group B	26.90±2.77	28.75±2.09	6.32, p=0.0001, S
Student’s unpaired t-test	3.34, p=0.002, S	5.23, p=0.0001, S	

**Figure 9:**
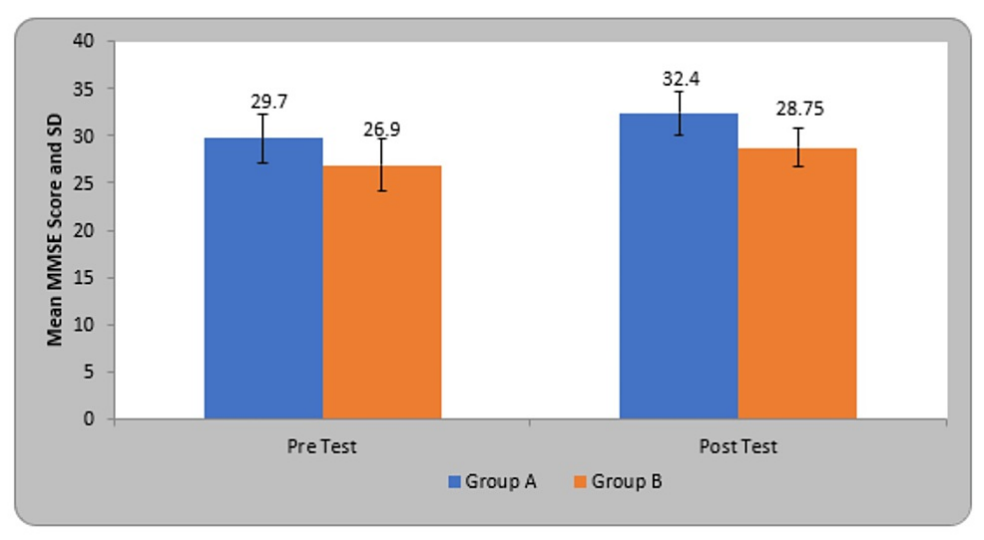
Comparison of MMSE score in two groups. MMSE - mini-mental state examination

The experimental group (Group A) is denoted by blue color and the Control group (Group B) by orange color (Figure 9).

In modified Ashworth Scale (MAS) scoring, for participants of group A, the pre-treatment, and post-treatment X2 value was 24 and the p-value was 0.0001. In group B, the pre-treatment, and post-treatment X2 value was 15.96 and the p-value was 0.0012. On comparison between both group A and group B, the X2 value was 44.85 while the p-value was 0.00001, which is statistically significant (Table 6).

**Table 6:** Distribution of children as per the MAS Score. MAS - Modified Ashworth Scale

MAS Score	Group A		Group B		א2-value
	Pre-Test	Post Test	Pre-Test	Post Test	
0	2(10%)	8(40%)	1(5%)	4(20%)	44.85 P=0.00001, S
1	2(10%)	8(40%)	3(15%)	7(35%)	
2	15(75%)	0(0%)	13(65%)	1(5%)	
1+	1(5%)	4(20%)	3(15%)	8(40%)	
Total	20(100%)	20(100%)	20(100%)	20(100%)	
א2-value	24		15.96		
p-value	0.0001, S		0.0012, S		

**Figure 10:**
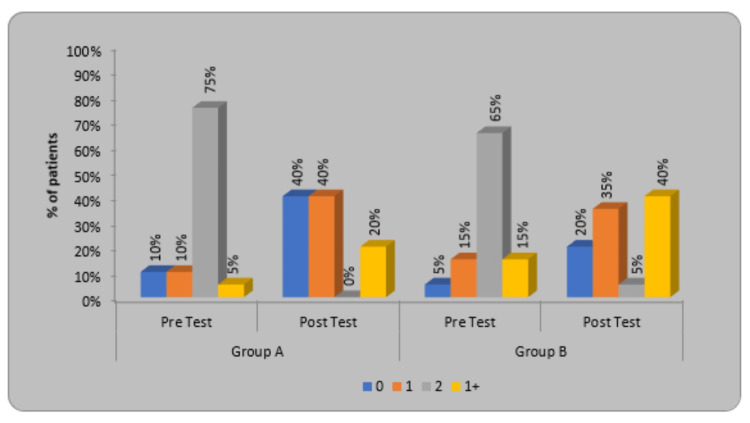
Distribution of children according to MAS score. MAS - Modified Ashworth Scale

The graph in Figure 10 shows the percentage of patients, in particular, MAS grade pre-test and post-test. Grades 0 and 1 are denoted by blue and orange color while grades 2 and 1+ are denoted by grey and yellow color.

## Discussion

This research was directed to review the efficacy of SIT on functional mobility in children with spastic diplegic CP. A difference in the GMFCS scoring shows a change in the capacity to perform gross motor skills. MAS indicates an improvement in muscle tone. An essential goal of therapeutic treatment for individuals with CP is getting them to perform proper exercises and take part in everyday routines. Preparing the program should take into account whether it is possible to implement these components in a clinical context.

In this study, a whole of 40 children of CP was assessed and all fulfilled the inclusion criteria. Forty children were divided into each group. Group A (Experimental group) was given SIT for 25 minutes and conventional physiotherapy for 20 minutes while Group B (Control group) was given 45 minutes of conventional physiotherapy only.

Clutterbuck has demonstrated a study that says that Active exercise therapy can improve motor function in CP children. They say that gross motor activity is a very usual and efficient therapy for CP children [21].

The inference of the study done by Booth et al. shows that functional gait training will enhance the walking ability of a child with CP [22]. Likewise, Dewar et al.'s study suggests that utilizing exercise-based therapy is effective in improving postural stability in CP children [23].

A study done by Chaovalit et al. shows that sit-to-stand activity protocol for CP children enhanced the sit-to-stand ability and resulted in a small development in self-care and motor function [24].

The evidence for SIT supporting changes in functioning and involvement emerges from research on young CP children. According to Kashefimerhr et al., occupational therapy programs utilizing SIT result in a child's non-functional behavioral interventions, vocabulary, abilities, cognition and physical performance, and environment adaption all improving significantly [13].

Moreover, current research by Drobnyk et al. raises the possibility that SIT can help children with Rett Syndrome (RTT) by increasing their rate of grasping. Given how profoundly incapacitating RTT is, even a minor change would be quite helpful [14].

Lee stated the link between motor function and the elements of functioning like activities and participation of children with spastic CP. They concluded that it is essential to consider, the physical abilities as well as limitations in functioning while treating CP children [25].

GMFCS was one of the outcome measures taken to evaluate the difference between pre-treatment and post-treatment. According to the study done by Leeuw et al. on the factors that influence change in self-care and mobility in CP children, it was concluded that GMFCS level as well as intellectual impairment for self-care, is one of the important and appropriate predictors for mobility capabilities [26].

In a study done by Pavãoa et al, a correlation between sensory processing and the activity performance in CP children of levels I-II on GMFCS is stated. The Sensory Profile (SP) and Pediatric Evaluation of Disability Inventory (PEDI) were also used for assessment. The inference was that sensory processing is linked to the ability of a CP child to do everyday tasks as well as social communication [27].

Bumin and Kayihan studied the efficacy of two sensory-integration programs for spastic diplegic CP children. Forty-one children were randomly allotted to three groups. First and second groups were given individual, and group sensory perceptual motor (SPM) training and third group received a home program only. Ayres Southern California Sensory Integration Test and Physical Ability Test were used for assessment. It was concluded that children with CP benefit from individual as well as group SPM training programs [28].

Mahaseth and Choudhary conducted a study on SIT versus traditional physical therapy in CP children on gross motor function. Thirty children were divided into two groups. One group received SIT with traditional Physiotherapy Exercises and the other group received traditional physical therapy only. Gross motor function measure (GMFM) and SSP were used for assessment before and after the therapy. And SIT in adjunct to conventional physiotherapy appeared to be more efficient and useful in the development of motor function in CP children [12].

With the data mentioned, the present study and the statistical analysis say that CP children have shown a notable change in functional mobility after receiving SIT along with conventional physiotherapy. Hence, the evidence suggests that the SIT is found to be an effective therapy for CP children to enhance functional mobility when given along with conventional physiotherapy.

SIT can be used to improve functional mobility in CP children. In everyday clinical practice, it can be a useful therapy to encourage more active participation from children. For future studies, more high-quality Randomized controlled trials can be performed by subgrouping the children into other types of CP, thus helping researchers to see the effect of SIT on them.

Limitations

Children suffering from spastic diplegia were only considered. The study can be done with long-term follow-up. The study can be done with a longer treatment duration. Individuals of the elder age group can be studied.

## Conclusions

SIT increases appropriate adaptive reactions to sensory inputs, improves focus, and encourages social relationships, all of which have a good effect on the child's response to sensation. The main objective of SIT is to enhance the organization, integration, and motor planning functions of the nervous system. According to this study, it has been concluded that conventional physiotherapy is efficient, but SIT is more effective in enhancing functional mobility in children with spastic diplegia.

## References

[1] Gillani SF, Rafique A, Taqi M, Chatta MA, Masood F, Ahmad Blouch T, Awais SM (2021). Effectiveness of treatment in children with cerebral palsy. Cureus.

[2] Murphy CC, Yeargin-Allsopp M, Decouflé P, Drews CD (1993). Prevalence of cerebral palsy among ten-year-old children in metropolitan Atlanta, 1985 through 1987. J Pediatr.

[3] Bax M, Goldstein M, Rosenbaum P (2005). Proposed definition and classification of cerebral palsy, April 2005. Dev Med Child Neurol.

[4] Thorpe D (2009). The role of fitness in health and disease: status of adults with cerebral palsy. Dev Med Child Neurol.

[5] Papavasiliou AS (2009). Management of motor problems in cerebral palsy: a critical update for the clinician. Eur J Paediatr Neurol.

[6] Kim JH, Choi YE (2017). The effect of task-oriented training on mobility function, postural stability in children with cerebral palsy. Korean Soc Phys Med.

[7] Vincer MJ, Allen AC, Joseph KS, Stinson DA, Scott H, Wood E (2006). Increasing prevalence of cerebral palsy among very preterm infants: a population-based study. Pediatrics.

[8] Stanley FJ (1992). Survival and cerebral palsy in low birthweight infants: implications for perinatal care. Paediatr Perinat Epidemiol.

[9] Kuban KC, Leviton A (1994). Cerebral palsy. N Engl J Med.

[10] (2022). Fenichel’s clinical pediatric neurology. https://www.elsevier.com/books/fenichel.

[11] Lane SJ, Mailloux Z, Schoen S (2019). Neural foundations of Ayres sensory integration(®). Brain Sci.

[12] Mahaseth PK, Choudhary A (2021). Sensory integration therapy verses conventional physical therapy among children with cerebral palsy on gross motor function - a comparative randomized controlled trial. Ann Romanian Soc Cell Biol.

[13] Kashefimehr B, Kayihan H, Huri M (2018). The effect of sensory integration therapy on occupational performance in children with autism. OTJR (Thorofare N J).

[14] Drobnyk W, Rocco K, Davidson S, Bruce S, Zhang F, Soumerai SB (2019). Sensory integration and functional reaching in children with Rett syndrome/Rett-related disorders. Clin Med Insights Pediatr.

[15] Schaaf R, Blanche EI (2011). Comparison of behavioral intervention and sensory-integration therapy in the treatment of challenging behavior. J Autism Dev Disord.

[16] Guardado KE, Sergent SR (2023). Sensory Integration. https://pubmed.ncbi.nlm.nih.gov/32644581/.

[17] Kilroy E, Aziz-Zadeh L, Cermak S (2019). Ayres theories of autism and sensory integration revisited: what contemporary neuroscience has to say. Brain Sci.

[18] Schaaf R, Mailloux Z (2015). Clinician’s Guide for Implementing Ayres Sensory Integration: Promoting Participation for Children With Autism. https://www.worldcat.org/title/clinicians-guide-for-implementing-ayres-sensory-integration-promoting-participation-for-children-with-autism/oclc/910656966.

[19] Randell E, McNamara R, Delport S (2019). Sensory integration therapy versus usual care for sensory processing difficulties in autism spectrum disorder in children: study protocol for a pragmatic randomised controlled trial. Trials.

[20] Himmelmann K, Horber V, Sellier E, De la Cruz J, Papavasiliou A, Krägeloh-Mann I (2020). Neuroimaging patterns and function in cerebral palsy-application of an MRI classification. Front Neurol.

[21] Clutterbuck G, Auld M, Johnston L (2019). Active exercise interventions improve gross motor function of ambulant/semi-ambulant children with cerebral palsy: a systematic review. Disabil Rehabil.

[22] Booth AT, Buizer AI, Meyns P, Oude Lansink IL, Steenbrink F, van der Krogt MM (2018). The efficacy of functional gait training in children and young adults with cerebral palsy: a systematic review and meta-analysis. Dev Med Child Neurol.

[23] Dewar R, Love S, Johnston LM (2015). Exercise interventions improve postural control in children with cerebral palsy: a systematic review. Dev Med Child Neurol.

[24] Chaovalit S, Dodd KJ, Taylor NF (2021). Sit-to-stand training for self-care and mobility in children with cerebral palsy: a randomized controlled trial. Dev Med Child Neurol.

[25] Lee BH (2017). Relationship between gross motor function and the function, activity and participation components of the International Classification of Functioning in children with spastic cerebral palsy. J Phys Ther Sci.

[26] de Leeuw MJ, Schasfoort FC, Spek B, van der Ham I, Verschure S, Westendorp T, Pangalila RF (2021). Factors for changes in self-care and mobility capabilities in young children with cerebral palsy involved in regular outpatient rehabilitation care. Heliyon.

[27] Pavão SL, Lima CR, Rocha NA (2021). Association between sensory processing and activity performance in children with cerebral palsy levels I-II on the gross motor function classification system. Braz J Phys Ther.

[28] Bumin G, Kayihan H (2001). Effectiveness of two different sensory-integration programmes for children with spastic diplegic cerebral palsy. Disabil Rehabil.

